# Supplementary material for: Computational predictions of hydrogen-assisted fatigue crack growth
Source: Int J Hydrogen Energy. 2024 Jun 27;72:315–25. doi: 10.1016/j.ijhydene.2024.05.264 (PMC13068105; doi:10.1016/j.ijhydene.2024.05.264)
Supplement: MMC S1 — Additional details of numerical implementation. [file mmc1.pdf]

Supplementary material

Computational predictions of hydrogen-assisted fatigue crack growth

Chuanjie Cui[a,b], Paolo Bortot[c], Matteo Ortolani[c], Emilio Martínez-Pañeda[a,b,*]

[a] *Department of Engineering Science, University of Oxford, Oxford OX1 3PJ, UK*
[b] *Department of Civil and Environmental Engineering, Imperial College London, London SW7 2AZ, UK*
[c] *Tenaris, Dalmine 24044, Italy*

Numerical implementation

For completeness and reproducibility, we proceed to provide additional details of the numerical implementation. The weak form of the governing equations for displacements $\mathbf{u}$, phase field order parameter ϕ, and hydrogen content C can be formulated as

$$\int_\Omega \left\{ \left[g(\phi) + \varkappa \right] \boldsymbol{\sigma}_0 : \text{sym}\, \nabla \delta \mathbf{u} \right\} \, \text{d}V + \int_{\partial\Omega} \mathbf{T} \cdot \delta \mathbf{u} \, \text{d}S = 0, \tag{1}$$

$$\int_\Omega \left\{ g'(\phi) \mathcal{H} \delta\phi + G_c f_{\text{F}}(\bar{\alpha}) f_{\text{H}}(C) \left[\frac{\phi}{\ell} \delta\phi - \ell \nabla\phi \nabla\delta\phi \right] \right\} \text{d}V = 0, \tag{2}$$

$$\int_\Omega \left[\left(\frac{1}{D} \frac{dC}{dt} \right) \delta C + \nabla C \nabla \delta C - \left(\frac{\bar{V}_H C}{R_g T} \nabla \sigma_H \right) \nabla \delta C \right] \text{d}V = 0. \tag{3}$$

Here $\varkappa = 1 \times 10^{-5}$ is a small, positive constant used to prevent ill-conditioning of the system when $\phi = 1$ and $\mathcal{H}$ is a history variable field introduced to ensure the irreversibility of crack propagation. The latter is defined as

$$\mathcal{H} = \max_{\tau \in [0,t]} \psi_0(\boldsymbol{\varepsilon}(\tau)). \tag{4}$$

Next, choices are made in relation to the finite element discretisation. Making use of Voigt notation, the primal kinematic variables are discretised in terms of their nodal values $\mathbf{u}_i = \{u_x, u_y, u_z\}^T$, ϕ_i, and C_i at node i as

$$\mathbf{u} = \sum_{i=1}^{m} \mathbf{N}_i \mathbf{u}_i, \quad \phi = \sum_{i=1}^{m} N_i \phi_i, \quad C = \sum_{i=1}^{m} N_i C_i, \tag{5}$$

*Corresponding author.
Email address: emilio.martinez-paneda@eng.ox.ac.uk (Emilio Martínez-Pañeda)

where m is the total number of nodes per element, N_i is the shape function associated with node i, and $\mathbf{N}_i$ is the shape function matrix, a diagonal matrix with N_i as components. Accordingly, the corresponding gradient quantities can be discretised by

$$\boldsymbol{\varepsilon} = \sum_{i=1}^{m} \boldsymbol{B}_i \mathbf{u}_i, \quad \nabla \phi = \sum_{i=1}^{m} \mathbf{B}_i \phi_i, \quad \nabla C = \sum_{i=1}^{m} \mathbf{B}_i C_i, \tag{6}$$

with $\boldsymbol{B}_i$ and $\mathbf{B}_i$ being the spatial derivatives of the shape functions.

The residuals can be readily derived from the weak form Eqs. (1)-(3) using the finite element discretisation (5)-(6)

$$\mathbf{r}_i^{\mathbf{u}} = \int_\Omega [g(\phi) + \varkappa] (\mathbf{B}_i)^T \boldsymbol{\sigma}_0 \, \mathrm{d}V - \int_{\partial\Omega} (\mathbf{N}_i)^T \mathbf{T} \, \mathrm{d}S, \tag{7}$$

$$r_i^{\phi} = \int_\Omega \left\{ g'(\phi) N_i \mathcal{H} + f_{\mathrm{F}}(\bar{\alpha}) f_{\mathrm{H}}(C) \, G_c \left(\frac{\phi}{\ell} N_i + \ell \, (\mathbf{B}_i)^T \nabla \phi \right) \right\} \mathrm{d}V, \tag{8}$$

$$r_i^{C} = \int_\Omega \left[\left(\frac{1}{D} \frac{dC}{dt} \right) N_i^T + \mathbf{B}_i^T \nabla C - \mathbf{B}_i^T \left(\frac{\bar{V}_H C}{R_g T} \nabla \sigma_H \right) \right] \mathrm{d}V. \tag{9}$$

Subsequently, we obtain the consistent tangent stiffness matrices $\boldsymbol{K}$ by differentiating the residuals with respect to the incremental nodal variables as follows

$$\boldsymbol{K}_{ij}^{\mathbf{u}} = \frac{\partial \mathbf{r}_i^{\mathbf{u}}}{\partial \mathbf{u}_j} = \int_\Omega \left\{ [g(\phi) + \varkappa] (\boldsymbol{B}_i)^T \boldsymbol{C}_0 \boldsymbol{B}_j \right\} \mathrm{d}V, \tag{10}$$

$$\mathbf{K}_{ij}^{\phi} = \frac{\partial r_i^{\phi}}{\partial \phi_j} = \int_\Omega \left\{ \left(g''(\phi) \mathcal{H} + f_{\mathrm{F}}(\bar{\alpha}) f_{\mathrm{H}}(C) \frac{G_c}{\ell} \right) N_i N_j \; + f_{\mathrm{F}}(\bar{\alpha}) f_{\mathrm{H}}(C) \, G_c \ell \, (\mathbf{B}_i)^T \mathbf{B}_j \right\} \mathrm{d}V, \tag{11}$$

$$\boldsymbol{K}_{ij}^{C} = \frac{\partial r_i^{C}}{\partial C_j} = \int_\Omega \left(\frac{1}{D \, \mathrm{d}t} \mathbf{N}_i^T \mathbf{N}_j + \mathbf{B}_i^T \mathbf{B}_j - \mathbf{B}_i^T \frac{\bar{V}_H}{R_g T} \nabla \sigma_H N_j \right) \mathrm{d}V. \tag{12}$$

An important aspect to consider is that providing accurate fatigue crack growth predictions necessarily requires resolving the interaction of a propagating crack with the environment. As such, one should capture how the H_2 gaseous environment quickly enters into contact with the newly created crack surfaces. To achieve this, we make use of a so-called penalty approach [1, 2], whereby the hydrogen concentration is forced to equal that of the environment (C_{env}) in damaged regions. This requires re-writing the residual and stiffness matrix terms of the hydrogen concentration, as follows

$$r_i^{C} = \int_\Omega \left[\left(\frac{1}{D} \frac{dC}{dt} + k_p \left(C - C_{\mathrm{env}} \right) \langle 4\phi - 3 \rangle \right) N_i^T + \mathbf{B}_i^T \nabla C - \mathbf{B}_i^T \left(\frac{\bar{V}_H C}{R_g T} \nabla \sigma_H \right) \right] \mathrm{d}V, \tag{13}$$

$$\boldsymbol{K}_{ij}^{C} = \frac{\partial r_i^{C}}{\partial C_j} = \int_\Omega \left(\frac{1}{D \, \mathrm{d}t} \mathbf{N}_i^T \mathbf{N}_j + \mathbf{N}_i^T \mathbf{N}_j k_p \langle 4\phi - 3 \rangle + \mathbf{B}_i^T \mathbf{B}_j - \mathbf{B}_i^T \frac{\bar{V}_H}{R_g T} \nabla \sigma_H N_j \right) \mathrm{d}V, \tag{14}$$

with k_p being a penalty term chosen to be sufficiently large so as to enforce $C = C_{\text{env}}$ in the cracked regions.

Finally, the linearised finite element system can be expressed as

$$\begin{bmatrix} \boldsymbol{K}^{\mathbf{u}} & \mathbf{0} & \mathbf{0} \\ \mathbf{0} & \mathbf{K}^{\phi} & \mathbf{0} \\ \mathbf{0} & \mathbf{0} & \mathbf{K}^{C} \end{bmatrix} \begin{bmatrix} \mathbf{u} \\ \boldsymbol{\phi} \\ C \end{bmatrix} = \begin{bmatrix} \mathbf{r}^{\mathbf{u}} \\ \boldsymbol{r}^{\phi} \\ \boldsymbol{r}^{C} \end{bmatrix} \tag{15}$$

The modelling framework is implemented in the commercial finite element package ABAQUS via a user element subroutine (UEL), and the finite element system is solved using time parametrisation and an incremental-iterative scheme in conjunction with the Newton–Raphson scheme. To guarantee accuracy, the solutions $\mathbf{u}$, ϕ, and C are obtained by a monolithic solution scheme. Computation times can be accelerated by using a cycle jump strategy, as proposed by Kristensen *et al.* [3], whereby the fatigue history variable $\bar{\alpha}$ at a time t can be reformulated as follows

$$\bar{\alpha}_t = \bar{\alpha}_{t-\Delta N \Delta t} + \Delta N \Delta \bar{\alpha}, \tag{16}$$

where ΔN denotes the range of loading cycles over which the change in fatigue damage is assumed to be similar. A sensitivity study is conducted to make sure that no noticeable inaccuracies are introduced by the use of this cycle jump strategy.

References

[1] Y. Renard, K. Poulios, GetFEM: Automated FE modeling of multiphysics problems based on a generic weak form language, ACM Transactions on Mathematical Software (TOMS) 47 (1) (2020) 1–31.

[2] E. Martínez-Pañeda, Z. D. Harris, S. Fuentes-Alonso, J. R. Scully, J. T. Burns, On the suitability of slow strain rate tensile testing for assessing hydrogen embrittlement susceptibility, Corrosion Science 163 (2020) 108291.

[3] P. K. Kristensen, A. Golahmar, E. Martínez-Pañeda, C. F. Niordson, Accelerated high-cycle phase field fatigue predictions, European Journal of Mechanics - A/Solids 100 (2023) 104991.
